# Ubiquitin over-expression phenotypes and ubiquitin gene molecular misreading during aging in Drosophila melanogaster

**DOI:** 10.18632/aging.100278

**Published:** 2011-03-12

**Authors:** Nicholas Hoe, Chung M. Huang, Gary Landis, Marian Verhage, Daniel Ford, Junsheng Yang, Fred W. van Leeuwen, John Tower

**Affiliations:** ^1^ Molecular and Computational Biology Program, Department of Biological Sciences, University of Southern California, Los Angeles, CA 90089-2910; ^2^ Netherlands Institute for Neuroscience, 1105 BA Amsterdam, The Netherlands; ^3^ Maastricht University, Department of Neuroscience, 6229ER Maastricht, The Netherlands

**Keywords:** Aging, Alzheimer's disease, ubiquitin, frameshift, development, life span

## Abstract

Molecular Misreading (MM) is the inaccurate conversion of genomic information into aberrant proteins. For example, when RNA polymerase II transcribes a GAGAG motif it synthesizes at low frequency RNA with a two-base deletion. If the deletion occurs in a coding region, translation will result in production of misframed proteins. During mammalian aging, misframed versions of human amyloid precursor protein (hApp) and ubiquitin (hUbb) accumulate in the aggregates characteristic of neurodegenerative diseases, suggesting dysfunctional degradation or clearance. Here cDNA clones encoding wild-type hUbb and the frame-shifted version hUbb^+1^ were expressed in transgenic Drosophila using the doxycycline-regulated system. Misframed proteins were abundantly produced, both from the transgenes and from endogenous Drosophila ubiquitin-encoding genes, and their abundance increased during aging in whole-fly extracts. Over-expression of wild-type hUbb, but not hUbb^+1^, was toxic during fly development. In contrast, when over-expressed specifically in adult flies, hUbb^+1^ caused small decreases in life span, whereas hUbb was associated with small increases, preferentially in males. The data suggest that MM occurs in Drosophila and that the resultant misframed proteins accumulate with age. MM of the ubiquitin gene can produce alternative ubiquitin gene products with different and sometimes opposing phenotypic effects.

## INTRODUCTION

The accurate read-out of genomic information into functional proteins is of critical importance to cellular homeostasis, and a significant disruption can lead to cell death [1, 2]. It has been hypothesized that a loss of fidelity in information flow could contribute to aging through a feed-forward loop or “error catastrophe” in which errors lead to an increasing frequency of errors [3]. Attempts to detect error catastrophe at the translational level during aging have generally been unsuccessful [4, 5], however error catastrophe could be rare, or the cells short-lived, or could occur at some other level of gene expression. Aging in several cell types and species is associated with a progressive loss of nuclear genome integrity and structure that could potentially reduce fidelity of information flow from the nucleus [6-8]. In addition, aging is characterized by significant changes in gene expression, including the tissue-specific induction of oxidative stress response genes and heat shock proteins (Hsps), and these gene expression changes may represent a response to mitochondrial malfunction, oxidative stress and proteotoxicity [9-13].

One aging-related alteration in information flow was discovered in the Brattleboro rat, which has a recessive form of diabetes insipidus (DI) due to a frame-shift mutation in the vasopressin precursor (VP) gene [14]. The DI mutation is a single nucleotide deletion and causes production of an abnormal (misframed) protein and loss of immunoreactivity. Surprisingly, it was found that in brain sections from rats homozygous for the DI mutation, rare solitary magnocellular neurons stained positively for VP, and their number increased with age. DNA and cDNA sequencing revealed that these revertant cells resulted from a process termed “Molecular Misreading” (MM), in which RNA polymerase inaccurately transcribes the DNA template [15]. One type of MM can occur when RNA polymerase II transcribes a GAGAG motif. The polymerase appears to sometimes “skip” 2 bases of coding sequence and generate RNA with a dinucleotide deletion. If the sequence is located in the gene's coding region, translation of the aberrant RNA can result in production of frame-shifted proteins. In the case of the Brattleboro rat's VP gene, MM at GAGAG hotspots restored the normal reading frame in the C-terminus and the production of immunoreactive protein [16]. Strikingly the same deletions were found to occur in transcripts from the wild-type rat and human VP genes, and because these transcripts would encode non-functional proteins it suggests that MM might have negative consequences during aging. Consistent with this idea, the human amyloid precursor protein (hApp) and human ubiquitin-B (hUbb) genes both have coding-region GAGAG hotspots, and the frame-shifted proteins (hApp^+1^ and hUbb^+1^) have been found associated with the neuritic plaques, neuropil threads, and neurofibrillary tangles characteristic of Alzheimer's disease (AD) [17, 18]. These mRNA deletions were independently confirmed, and MM events were identified at additional short simple repeat motifs in the hApp and hUbb transcripts [19]. In nervous tissue from both AD and Down Syndrome patients where hApp^+1^ and hUbb^+1^ proteins were present, the concentration of the corresponding deleted mRNAs was not detectably increased, suggesting that in these cases abnormal protein accumulation results from a defect in clearance or turnover of abnormal proteins [20].

Ubiquitin is normally ligated to other proteins in the cell as a monomer or polymer to regulate their activity and/or entry into proteasomal and other degradation pathways [21]. Misframed hUbb (hUbb^+1^) has an extended C terminus that alters its cross-linking properties, and in a dose-dependent way hUbb^+1^ can cause proteasome malfunction and apoptotic cell death in mammalian cells [9, 22-24]. Similarly, in yeast cells, Ub^+1^ has been shown to inhibit proteasome function and enhance toxic protein aggregation and cell death [25, 26]. In humans the hUbb^+1^ has been found associated with the abnormal protein inclusions that characterize several human disease states in addition to AD, including “tauopathies” [27], polyglutamine diseases [28], alcoholic cirrhosis [29], and inclusion body myositis [30], suggesting it may be a general marker for proteasomal malfunction [27].

The accumulation of inactive enzymes in nematodes was among the first molecular characteristics of aging identified [31]. Since then aging across many species and tissues has been shown to be associated with the accumulation of proteins that are conformationally altered, oxidatively and hydrolytically damaged, glycated and cross-linked [32-36]. The ubiquitin-regulated protein degradation pathways mediate the turnover of many such damaged proteins, and ubiquitin expression is increased in response to heat and oxidative stress and during aging in various mammalian and Drosophila tissues [37, 38].

Ubiquitin regulates several critical processes in addition to protein degradation, including chromatin remodeling [39], gene silencing involving mono-ubquitylation of H2A [40-42], membrane trafficking [43], and targeting of proteins to specific subcellular organelles such as the mitochondria [9, 44-46]. Efficient proteosomal degradation requires a multi-ubiquitin chain [47], while proteins that are mono-ubiquitylated on one or more lysine residues are stable. In addition to the proteosomal pathway, ubiquitin also regulates protein degradation via the lysosome and autophagosome pathways, through mechanisms affected by ubiquitin chain length and linkage type [48, 49]. Free monomeric ubiquitin is rare in the cell, and competition for this limited pool may be a mechanism for coordinating the various ubiquitin-regulated processes. The histones in the chromatin of the nucleus are abundantly ubiquitylated, and treatment of cells with proteasome inhibitors or heat shock depletes ubiquitin from the histones and causes changes in gene expression and a more condensed chromatin conformation [39, 50, 51].

It has become increasingly apparent that cells use a variety of methods to maximize the coding potential of nucleic acids, including alternate and over-lapping reading frames and RNA splicing and editing. RNAs encoding misframed proteins are often degraded by the nonsense-mediated-decay pathway (NMD), however, human Ubb^+1^ escapes from NMD because it has no downstream intron [52]. In Drosophila the NMD pathway is required for larval viability and affects RNA abundance for numerous wild-type genes, including *ornithine decarboxylase antizyme* and *transformer*[53, 54]. While MM might represent deterioration in fidelity of information flow, another possibility is that MM might be a regulated process or oxidative stress response [24] that the cell uses to generate alternate gene products with possibly different functions. To facilitate the study of MM and its possible relationship to aging, it was asked whether MM or related processes could be observed in the model organism *Drosophila melanogaster*.

**Figure 1.**
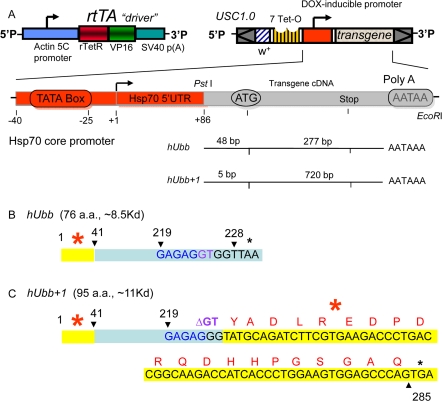
Diagram of transgenic constructs (**A**) The “Tet-on” conditional transgene expression system. The rtTA transgenic construct (or “driver”) contains the tissue-general actin5C promoter driving expression of the artificial transcription factor rtTA. The target constructs were generated by cloning the indicated cDNA fragments downstream of the DOX-inducible promoter in the USC1.0 vector between the unique PstI and EcoRI sites. The number of bases present upstream and downstream of the A residue of the ATG start codon for normal translation are indicated for each cDNA insert. The rtTA protein will bind to the 7 Tet-O sites in the target construct promoter and activate transcription only in the presence of DOX. (**B**) Diagram of the hUbb construct. The number 1 indicates the A of the normal ATG start codon for translation of hUbb, and the stop codon is indicated by a black asterisk. (**C**) Diagram of the hUbb^+1^ construct. The GAGAG hotspot for MM is indicated in blue, and the GT dinucleotide is indicated in purple. Note that in the hUbb^+1^ construct the GT dinucleotide has been deleted so that this construct constitutively encodes hUbb^+1^ protein. The amino acid sequence of the peptide used to generate the hUbb^+1^ antibody is indicated in red. The independently derived transgenic strains are given names comprised of the name of the inserted construct (e.g., hUbb or hUbb^+1^) followed by a unique number in brackets indicating the particular independent transgenic line.

## RESULTS

### Generation and conditional expression of transgenic constructs

**Figure 2.**
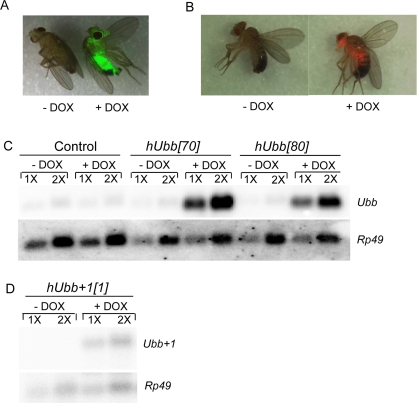
Conditional transgene expression Flies of the indicated genotypes were cultured for one week on food supplemented +/− DOX, as indicated. (**A, B**) Doxycycline regulated expression of the TetO-GFP and TetO-DsRED reporters. GFP and DsRED images of live, CO_2_-anesthetized flies were generated using the LeicaMZFLIII fluorescence stereomicroscope, and are overlayed with the visible image. A. The rtTA(3)E2 driver was crossed to the TetO-GFP[8] reporter line. B. The TO-daughterless driver was crossed to the TetO-DsRED[26B] reporter line. (**C, D**) Northern analysis. Total RNA was isolated from 30 flies, quantified by spectrophotometer, and 5μg (1X) and 10μg (2X) amounts were loaded for each sample. The resultant blot was hybridized with the indicated gene-specific probes. (**C**) Control flies and hUbb transgenic fly strains. D. hUbb^+1^ transgenic fly strain.

To determine if MM could be studied in Drosophila, cDNA clones encoding wild-type and frame-shifted versions of the human ubiquitin protein were expressed in Drosophila using the conditional doxycycline(DOX)-regulated system (“Tet-on”) [55, 56]. In the DOX-regulated system, the control and experimental animals have identical genetic backgrounds, and transgene expression is induced in larvae or adults by feeding the drug DOX. In this way any possible toxic effects of the RNAs or proteins can be avoided or reduced, because expression should occur only in the presence of DOX. A human cDNA encoding the wild-type ubiquitin protein and a cDNA engineered with the appropriate dinucleotide deletion adjacent to the GAGAG motif were cloned downstream of the DOX-regulated promoter (Figure 1). These constructs were introduced into Drosophila using P element mediated transformation and multiple independent transgenic strains were generated for each construct. In all the experiments presented, the strains homozygous for the transgenic target constructs were crossed to the rtTA(3)E2 driver strain (or other driver strains, as indicated), to generate hybrid progeny containing both constructs. In the *rtTA* construct the powerful, tissue-general cytoplasmic actin (*actin5C*) promoter drives expression of the artificial transcription factor rtTA. Upon DOX feeding the rtTA protein undergoes a conformation change and binds to specific sequences (called TetO) in the target construct, thereby activating transgene expression in all tissues except for the germ-line; titration of DOX in the food yields a dose-dependent increase in transgene expression [56]. To control for possible effects of the drug, the rtTA(3)E2 line was crossed to non-transgenic fly strains (either Oregon-R wild-type or the w[1118] strain, as indicated) to generate hybrid progeny containing only the rtTA(3)E2 driver construct and no target construct (Control flies). As part of these experiments, target constructs encoding the fluorescent proteins eGFP and DsRED were generated to use as controls for the efficiency and tissue-specificity of transgene expression. Assay of these fluorescent reporter lines confirmed that the DOX-regulated system yields high-level, tissue-general transgene expression (Figure 2A, B), as had previously been demonstrated using reporters encoding β-galactosidase [55, 56]. Conditional (DOX-dependent) expression of the wild-type and misframed hUbb transgenes was confirmed at the level of RNA transcripts using Northern blots (Figure 2C-D).

The human ubiquitin-B gene encodes three direct repeats of ubiquitin protein that is subsequently processed into mature monomers. The GAGAG hotspot for MM is located at the 3' end of each repeat, such that MM causes an almost full-length ubiquitin moiety to be fused with part the next repeat in the +1 frame, thereby creating an altered ubiquitin protein with a C-terminal extension of 20 amino acids, called hUbb^+1^. The hUbb construct created here contains only the single 5'-most ubiquitin repeat, and is therefore designed to encode a wild-type hUbb monomer (Figure 1B). The hUbb^+1^ construct contains two hUbb repeats, with the appropriate dinucleotide deletion engineered at the GAGAG hotspot at the end of the first repeat, thereby constitutively encoding hUbb^+1^ (Figure 1C). However, note that the 5'-most repeat of the hUbb gene contains sequences at the 5' end (indicated in yellow with red asterisk in Figure 1B,C), which, if translated out of frame, could encode an epitope with partial homology to the bona fide +1 epitope located downstream of the GAGAG hotspot (see Discussion). The nucleotide sequences and translations of the hUbb and hUbb^+1^ construct transcripts are presented in Supplemental Materials (Supplemental Figure S1). The endogenous Drosophila ubiquitin-encoding genes include two polyubiquitin genes, DmUbi-p63E with 10 repeats, and DmUbi-p5E with 3 repeats [57], as well as fusions of ubiquitin to other coding sequences that are conserved in mammals [58].

### Western analysis of hUbb expression

**Figure 3.**
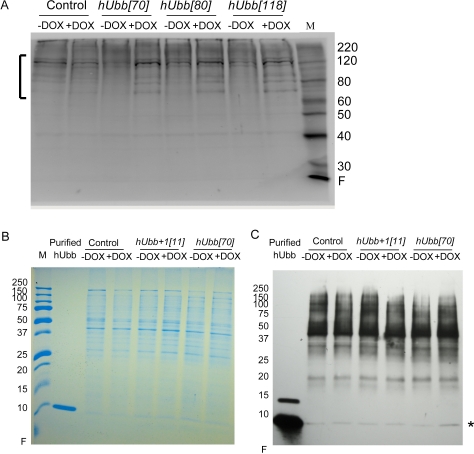
Western analysis of hUbb protein expression Total protein was isolated from 30 male flies, diluted as indicated, fractionated using SDS-PAGE and Western blotted. (**A**) Control flies and hUbb transgenic strain fly protein incubated with antibody specific for hUbb. The bracket indicates high MW species induced by DOX. (**B**) Control flies and transgenic strains expressing hUbb or hUbb^+1^, total protein stain. Purified hUbb protein monomer was run as control. (**C)** Control flies and transgenic strains expressing hUbb or hUbb^+1^, incubated with antibody specific for hUbb. Purified hUbb protein monomer is run as control (position indicated by asterisk). Samples are the same as shown in (**B**). The change in monomer abundance upon DOX treatment was determined using densitometry: control = 0.94; hUbb^+1^[11] = 0.98; hUbb[70] = 3.2 fold.

Western blot analysis with a specific antibody was used to assay for expression of the hUbb protein in flies. The human and Drosophila ubiquitin proteins are identical in amino acid sequence, so it was expected that antibody raised against hUbb would cross-react with endogenous Drosophila protein. Consistent with this expectation, the hUbb antibody recognized a series of protein bands in control fly extracts, including numerous high-MW species and a single band at the ~8.5Kd size calculated for monomeric ubiquitin (Figure 3A-C). Several abundant high-MW proteins recognized by the hUbb antibody are indicated by a bracket (Figure 3A). These species are interpreted to represent endogenous Drosophila ubiquitin ligated to various proteins in the cell. Importantly the abundance of these protein species was not altered by DOX treatment in the control flies, indicating that DOX itself does not have a detectable effect on ubiquitin expression. A similar pattern of high-molecular-weight species were also present in the extracts of transgenic flies where hUbb was being expressed, and notably the abundance of these species was induced by DOX in each of the three independent transgenic lines tested (Figure 3A). These results are consistent with DOX-dependent expression of hUbb from the transgenes that is then rapidly ligated to fly cellular proteins. Monomeric ubiquitin was found to be less abundant and more difficult to detect. A scarce and limiting pool of free ubiquitin has previously been suggested to explain the low abundance of ubiquitin monomers relative to multimers in mammalian cell culture studies[39]. By loading larger amounts of fly protein, and by employing a gradient gel to resolve small MW proteins from the gel front, monomeric ubiquitin could be detected at the expected ~8.5Kd size, and was confirmed by co-migration with purified monomeric ubiquitin (Figure 3 B, C; indicated by asterisk). As expected, the monomeric ubiquitin species was induced by DOX in the hUbb transgenic line (~ 3 fold increase), but not in the control flies or the flies expressing hUbb^+1^ (Figure 3C).

**Figure 4.**
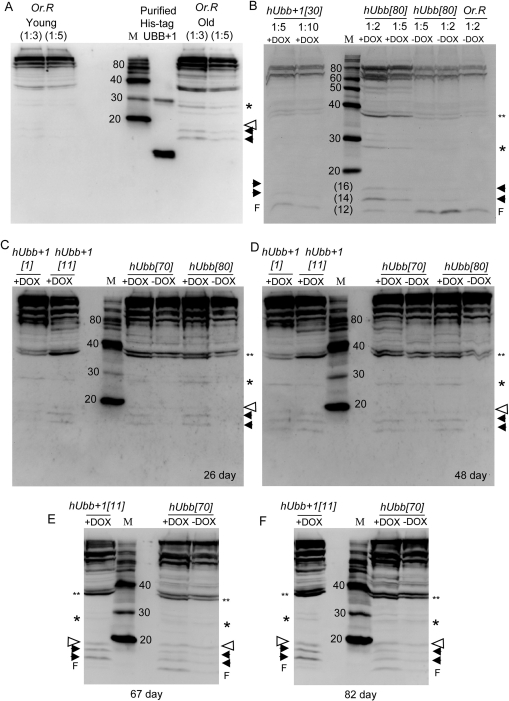
Western blot analysis using antibody specific for hUbb^+1^ Total protein was isolated from 30 male flies of the indicated genotypes, and 1/8 of the sample was assayed for the presence of protein that would be recognized by hUbb^+1^ antibody. Where indicated protein samples were diluted 1:2, 1:3, 1:5 or 1:10 to confirm sensitivity of the assay to relative protein concentrations. In panels **B-F** all samples are diluted 1:3. (**A**) Molecular weight markers were run alongside His-tagged hUbb^+1^ purified from *E. coli* cells as well as total protein isolated from 30 “young” (10 day old) and “old” (65 day old) male Oregon-R control flies, as indicated. (**B**) “Young” (10 day old) flies of the indicated genotypes. Note the hUbb[80] –DOX sample lanes contain cross-reacting material that is unresolved from the gel front (**F**), and is interpreted as degradation products. This material was not present in other hUbb[80] protein samples (see panels **C** and **D**). (**C**) Flies cultured +/− DOX for 26 days. (**D**) Flies cultured +/− DOX for 48 days. (**E**) Flies cultured +/− DOX for 67 days. (**F**) Flies cultured +/− DOX for 82 days. Where visible the gel protein front (**F**) is indicated. Solid arrowheads indicate two species of <20Kd, either of which might represent Ub^+1^ monomer, which has an expected size of ~11Kd. Open arrowhead indicates species at expected position for Ub^+1^ ligated to one Ub wild-type protein (^~^11Kd + ^~^8.5Kd = ^~^19.5Kd). Single asterisk indicates species at expected position for Ub^+1^ ligated to two Ub proteins (^~^ 11Kd + ^~^17Kd = ^~^28Kd). Double asterisk indicates species at expected position for Ub^+1^ ligated to three Ub proteins (^~^11Kd + ^~^25.5Kd = ^~^37Kd). Estimations of sizes of various species are presented in Supplemental Materials.

### Western analysis of Ub^+1^ expression and increase during aging

**Figure 5.**
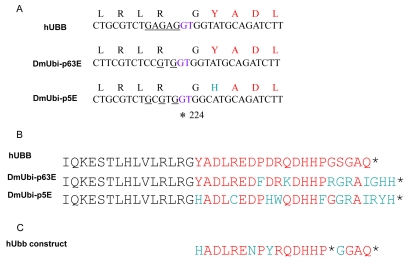
Comparison of human and Drosophila ubiquitin gene sequences (**A**) The GAGAG hotspot for MM in the human polyubiqitin-B gene (hUBB) is indicated by underline, and the GT dinucleotide deleted upon MM is indicated in purple, located at position +224 of the mRNA. Single letter amino acid code indicates the translation frame produced upon deletion of the GT dinucleotide. The corresponding region is indicated for the Drosophila polyubiqitin genes DmUbi-p63E and DmUbi-p5E. (**B**) The +1 epitope of the human Ubb^+1^ protein is indicated in red, alongside the corresponding regions of the predicted Drosophila Ub^+1^ proteins. (**C**) The potential +1 epitope encoded by the 5? sequences of the single hUbb repeat in the hUbb construct is presented. Translation of the entire hUbb construct transcript in each reading frame is presented in Supplemental Figure S1.

To determine if expression of the misframed (+1) version of the hUbb protein could be detected, antibody specific for hUbb^+1^ was used in Western blot assays. This antibody had been previously characterized and shown to be highly specific for hUbb^+1^[9, 17]. As expected this hUbb^+1^ antibody strongly recognized purified His-tagged hUbb^+1^ protein purified from *E. coli* cells (Figure 4A). Strikingly, the hUbb^+1^ antibody also recognized a complex pattern of bands in extracts of Oregon-R control flies that became more abundant with age, including large amounts of high-MW material, as well as several small species migrating at an apparent MW of <20Kd (Figure 4A). These species are interpreted to represent Ub^+1^ protein produced from the endogenous Drosophila Ub-encoding genes for two reasons: (i) the ubiquitin gene sequences are highly conserved between the human and the fly, such that the endogenous fly genes encode a Ub^+1^ protein similar to human (Figure 5), (ii) a similar pattern of DOX-inducible species was produced by both the hUbb^+1^ and hUbb transgenes (Figure 4B-F). The hUbb^+1^ transgene produced a series of bands that cross-reacted with the hUbb^+1^ antibody, both small MW species as well as higher MW species, and that increased in abundance with age of the flies (Figure 4B-F). This pattern of proteins was highly similar to that observed in the old Oregon-R control flies (Figure 4B), and also appeared to include several additional species. The calculated size for the Ub^+1^ monomer is ~11Kd, and this may correspond to one of the DOX-inducible species migrating at an apparent MW of <20Kd (indicated with black arrowheads in Figure 4; estimation of sizes is shown in Supplemental Figure S2), or alternatively the monomeric Ub^+1^ form may be of too low abundance to be detected. Ub^+1^ is itself known to be a target for (poly)ubiqitination by wild-type ubiqutin (monomeric MW ~8.5Kd), and notably a faint DOX-inducible species was present at the MW predicted for Ub^+1^ ligated to one ubiquitin moiety (~19.5Kd, indicated by an open arrowhead), as well as Ub^+1^ ligated to two ubiquitin proteins (~28Kd, indicated by single asterisk) and Ub^+1^ ligated to three ubiquitin proteins (~37Kd, indicated by double asterisk) (estimation of apparent MW is presented in Supplemental Figure 2).

Strikingly, the hUbb transgenic strains produced a similar series of bands whose abundance was induced by DOX and that cross-reacted with the hUbb^+1^ antibody (Figure 4B-F). These included small MW species similar to those described above, as well as a similar series of higher MW species. Notably, because the hUbb transgene used here encodes only one ubiquitin repeat (Figure 1A, B), a MM event at the GAGAG hotspot (position 219 of the ORF) will not produce hUbb^+1^ protein, because the GAGAG hotspot is located downstream of the relevant epitope in this construct (Figure 1B; epitope region indicated in yellow highlight and with red asterisk). Therefore the induced expression of the hUbb transgene must be altering the abundance of Ubb^+1^ protein species by undergoing a MM event at a location upstream of the epitope (see Figure 5C; Supplemental Figure S1), and/or because induced expression of hUbb increases the levels of the many abundant endogenous Ub^+1^ protein species, through ligation or other effects (see Discussion).

Multiple transgenic Drosophila strains were also generated using constructs designed to encode hApp and hApp^+1^ proteins (Supplemental materials). Expression of hApp protein could not be detected in adult male flies using these methods (Supplemental Figure S3D). However, DOX-dependent expression of hApp^+1^ protein was readily detected, using transgenes encoding hApp^+1^, as well as transgenes encoding wild-type hApp, and the hApp^+1^ protein became more abundant with age (Supplemental Figure S4), consistent with MM of the hApp construct.

### Phenotypic consequences of expression of hUbb and hUbb^+1^

**Figure 6.**
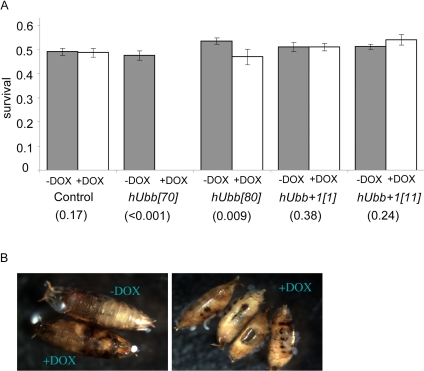
Effect of hUbb and hUbb^+1^ over-expression on developmental survival (**A**) Frequency of adult flies containing both the rtTA(3)E2 driver and the indicated target transgene that emerged from crosses where larval development was allowed to occur in the presence and absence of DOX, as indicated. Control flies contained no target gene. P values obtained by chi-square test are presented in parentheses. (**B**) Examples of the pupal-lethal phenotype resulting from hUbb[70] transgenic line cultured +/− DOX, as indicated.

It was next asked if expression of wild-type and +1 versions of hUbb transgenes would have phenotypic consequences for the flies. Over-expression of the highly-expressed hUbb[70] transgene during larval development was found to be lethal, and slightly reduced viability was associated with the less strongly expressing line hUbb[80] (Figure 6A). The lethality caused by hUbb over-expression was associated with a dramatic disruption of normal pupae structures and large melanotic inclusions indicative of extensive cell death (Figure 6B). Reduced survival and melanotic inclusions were also observed with another highly-expressing hUbb strain, hUbb[118D] (data not shown). In contrast there was no evidence of reduced survival or pupal abnormalities when the hUbb^+1^ transgenes were expressed during development, using a variety of drivers and multiple independent hUbb^+1^ transgenes (Figure 6A, and additional data not shown).

**Figure 7.**
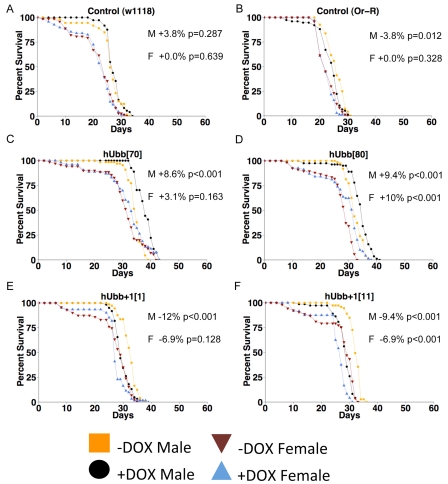
Life span assays The TO-daughterless driver line was crossed to the indicated transgenic strains, as well as to the w[1118] and Oregon-R strains to generate controls containing the driver but no target transgene. Adult life span was assayed at 29°C. (**A**) Control (w1118 cross). (**B**) Control (Oregon-R cross). (**C**) hUbb[70]. (**D**) hUbb[80]. E. hUbb^+1^[1]. (**F**) hUbb^+1^[11]. The percent change in median life span for males (M) and females (F) is presented in each panel, along with the p value obtained by log rank test. Additional life span data and statistical analyses are summarized in Table 1.

The same transgenes were over-expressed specifically in adult flies to assay for possible effects on life span. In the first experiments, the TO-daughterless driver was employed to yield tissue-general transgene expression, and life span was assayed at 29^0^C. Control flies were generated by crossing to the driver strain to either the wild-type Oregon-R strain or the w[1118] strain, to generate control flies containing the driver construct(s) but no target gene. In these control flies administration of DOX had no significant effect on life span, except for control (Oregon-R) males where life span was decreased by -3.8% (Figure 7 A, B). These data demonstrate that DOX itself does not generally have a significant effect on life span, and illustrate the background variation of the assay, which is typically within the range of +/−5%. When hUbb^+1^ was over-expressed in the adult flies, it was found to have small negative effects on survival, particularly in males (Figure 5E, F; data summarized in Table 1). In contrast, hUbb did not have these negative effects and instead was associated with slightly increased life span, again preferentially in males (Figure 5C, D). Three additional life span assays were conducted to determine if the increased life span caused by hUbb over-expression would be also be observed at 25^0^C. Small but variable increases in life span were again observed in males, ranging from 0-14%, whereas female life span was unchanged or slightly decreased (Table 1).

**Table 1:** Life span assay summary and statistical analyses

**TO-daughterless driver, 29^0^C, Males**								
**Genotype**	**RU486**	**Sample Size**	**Mean (SD)**	**Median**	**90% Mortality**	**Change in Mean (%)**	**Change in Median (%)**	**Log-Rank Test (p)**
*Control w[1118]*	−	73	26 (5)	26	32	5.3	3.8	0.287
	+	73	27 (3)	27	31			
*Control Or-R*	−	68	26 (3)	26	29	−7.4	−3.8	0.012
	+	76	24 (4)	25	28			
*hUbb+1(I)*	−	73	32 (3)	33	36	−8.1	−12	5.7E-5
	+	61	30 (3)	29	34			
*hUbb+1(II)*	−	73	32 (2)	32	34	-12	-9.4	4.4E-16
	+	73	28 (3)	29	31			
*hUbb[70]*	−	66	35 (3)	35	38	10	8.6	5.2E-6
	+	27	38 (3)	38	41			
*hUbb[80]*	−	75	32 (3)	32	36	5.5	9.4	3.1E-5
	+	82	34 (4)	35	39			
**TO-daughterless driver, 29^0^C, Females**								
**Genotype**	**RU486**	**Sample Size**	**Mean (SD)**	**Median**	**90% Mortality**	**Change in Mean (%)**	**Change in Median (%)**	**Log-Rank Test (p)**
*Control w[1118]*	−	73	21 (7)	24	28	−1.3	0	0.639
	+	69	22 (7)	24	28			
*Control Or-R*	−	73	23 (3)	22	27	−1.6	0	0.328
	+	69	22 (3)	22	26			
*hUbb+1(I)*	−	71	27 (7)	29	33	−0.4	−6.9	0.128
	+	61	27 (6)	27	32			
*hUbb+1(II)*	−	53	27 (7)	29	32	−5.8	−6.9	2.0E-4
	+	24	25 (6)	27	29			
*hUbb[70]*	−	70	31 (8)	32	40	3.8	3.1	0.163
	+	53	32 (8)	33	41			
*hUbb[80]*	−	67	27 (6)	29	32	5.5	10	2.0E-4
	+	69	28 (8)	32	34			
**rtTA(3)E2 driver, 25^0^C, Males, Experiment 1**								
**Genotype**	**RU486**	**Sample Size**	**Mean (SD)**	**Median**	**90% Mortality**	**Change in Mean (%)**	**Change in Median (%)**	**Log-Rank Test (p)**
*Control w[1118]*	−	130	89 (15)	92	102	5.1	4.3	3.5E-5
	+	121	93 (15)	96	108			
*hUbb[70]*	−	124	80 (10)	82	92	9.8	7.3	4.4E-7
	+	122	88 (7)	88	96			
*hUbb[80]*	−	130	83 (9)	84	92	7.4	7.1	1.8E-10
	+	132	89 (12)	90	100			
**rtTA(3)E2 driver, 25^0^C, Females, Experiment 1**								
**Genotype**	**RU486**	**Sample Size**	**Mean (SD)**	**Median**	**90% Mortality**	**Change in Mean (%)**	**Change in Median (%)**	**Log-Rank Test (p)**
*Control w[1118]*	−	113	83 (16)	84	94	2.6	4.8	0.023
	+	119	85 (16)	88	98			
*hUbb[70]*	−	118	77 (16)	84	90	4.8	−2.4	0.220
	+	126	81 (11)	82	92			
*hUbb[80]*	−	128	81 (14)	84	92	−7.8	−4.8	4.2E-4
	+	125	74 (18)	80	88			
**rtTA(3)E2 driver, 25^0^C, Males, Experiment 2**								
**Genotype**	**RU486**	**Sample Size**	**Mean (SD)**	**Median**	**90% Mortality**	**Change in Mean (%)**	**Change in Median (%)**	**Log-Rank Test (p)**
*Control w[1118]*	−	122	87 (14)	90	102	−1.2	2.2	0.357
	+	122	86 (18)	92	102			
*hUbb[70]*	−	124	95 (18)	100	112	1.3	−2.0	0.392
	+	123	97 (13)	98	112			
*hUbb[80]*	−	117	84 (16)	84	98	11.0	14.3	1.56E-6
	+	115	93 (17)	96	111			
**rtTA(3)E2 driver, 25^0^C, Females, Experiment 2**								
**Genotype**	**RU486**	**Sample Size**	**Mean (SD)**	**Median**	**90% Mortality**	**Change in Mean (%)**	**Change in Median (%)**	**Log-Rank Test (p)**
*Control w[1118]*	−	120	96 (14)	98	110	−7.7	−2.0	.910
	+	112	89 (26)	96	114			
*hUbb[70]*	−	123	96 (21)	102	110	6.36	0.0	0.000217
	+	120	102 (20)	102	116			
*hUbb[80]*	−	118	88 (23)	94	108	0.075	2.12	0.119
	+	116	88 (29)	96	114			
**rtTA(3)E2 driver, 25^0^C, Males, Experiment 3**								
**Genotype**	**RU486**	**Sample Size**	**Mean (SD)**	**Median**	**90% Mortality**	**Change in Mean (%)**	**Change in Median (%)**	**Log-Rank Test (p)**
*Control w[1118]*	−	116	93 (16)	96	110	5.24	4.17	0.00568
	+	119	98 (15)	100	114			
*hUbb[70]*	−	117	93 (13)	92	106	2.88	4.34	0.0136
	+	122	96 (14)	96	112			
*hUbb[80]*	−	97	92 (15)	92	107	3.88	6.52	0.153
	+	116	96 (11)	98	110			
**rtTA(3)E2 driver, 25^0^C, Females, Experiment 3**								
**Genotype**	**RU486**	**Sample Size**	**Mean (SD)**	**Median**	**90% Mortality**	**Change in Mean (%)**	**Change in Median (%)**	**Log-Rank Test (p)**
*Control w[1118]*	−	118	102 (14)	106	116	−1.15	−3.77	0.603
	+	121	101 (13)	102	114			
*hUbb[70]*	−	123	105 (8)	106	116	−3.43	−1.89	0.581
	+	123	102 (20)	104	118			
*hUbb[80]*	−	127	100 (19)	102	116	1.01	−0.98	.0407
	+	126	101 (14)	101	114			

## DISCUSSION

In the present study wild-type and misframed versions of hUbb protein were identified based on their apparent MW in SDS-PAGE gels, co-migration with proteins purified from *E. coli*, DOX-inducible expression from transgenic constructs, and cross-reactivity with specific antibodies. The Western blot analyses suggested that wild-type hUbb and misframed hUbb proteins were successfully expressed from the transgenes designed to encode these proteins. Notably the hUbb^+1^ species were more readily detected in extracts from old flies, supporting the connection between MM and aging. Expression of hApp protein could not be detected in adult male flies using our methods. However, DOX-dependent expression of hApp^+1^ protein was readily detected, using transgenes encoding hApp^+1^, as well as transgenes encoding wild-type hApp, and the hApp^+1^ protein became more abundant with age, consistent with MM of the hApp construct.

It was striking that the hUbb^+1^ antibody recognized a series of abundant endogenous protein species in control flies. The fact that several of these species appeared to co-migrate with DOX-inducible bands produced by the hUbb^+1^ transgene (and hUbb transgene) supported their identification as containing bona fide Ub^+1^ protein. This suggests that the endogenous Drosophila ubiquitin-encoding gene(s) are undergoing MM and producing abundant Ub^+1^ protein of various sizes, likely involving cross-linking to other cellular proteins such as ubiquitin, and moreover that these species become more abundant during aging. In the human Ubb and App genes, MM can occur at GAGAG hotspots as well as at other simple repeat motifs [19]. The endogenous Drosophila polyubiquitin genes contain only a partial match to the GAGAG hotspot, however, they do contain a conserved adjacent GT dinucleotide at position +224 of the corresponding mRNAs (Figure 5A), which if deleted would lead to production of a Ub^+1^ protein similar to that of humans (Figure 5B).

The ability of the hUbb transgene to produce DOX-inducible species that cross react with Ubb^+1^ antibody is consistent with possible MM of the hUbb transgenic construct, however these events cannot be occurring at the GAGAG hotspot as it is located only downstream of the relevant epitope in this construct (Figure 1B). One possibility is that one or more other DNA sequence elements located in the 5' end of the wild-type hUbb construct are leading to MM. However the nature of these possible MM events is not clear at this time, as the largest ORF containing the (+1) epitope in the hUbb construct does not contain an ATG start codon, and would encode a protein of only 45 amino acid residues (~ 5Kd) (Supplemental Figure S1). An alternative possibility, and the one that we favor, is that the DOX-inducible expression of hUbb is altering the abundance of the endogenous Drosophila Ub^+1^ species, either by affecting the expression and MM of the endogenous Drosophila ubiquitin genes, and/or by altering the stability and cross-linking of the abundant endogenous Drosophila Ub^+1^ protein species. For example, the hUbb protein expressed from the transgene is likely to ligate to the endogenous Drosophila Ub^+1^ proteins, thereby favoring the abundance of the heteromeric complexes (Figure 4).

One line of evidence in support of a phenotypic consequence for MM is the effect of the over-expressed genes. Ubiquitylation with the normal ubiquitin serves both pro-apoptotic and anti-apoptotic functions, depending upon the target and the cellular context [59]. The disruption and cell death observed here upon over-expression of hUbb during pupal development may indicate a pro-apoptotic phenotype useful for future studies. While high-level expression of hUbb was toxic to developing pupae, over-expression of hUbb^+1^ was not, consistent with different functions for the two proteins. Moreover, hUbb appeared to have small benefits for survival of adult male flies, while hUbb^+1^ was slightly toxic. To what extent endogenous Ub^+1^ might function in normal Drosophila cell physiology will be an interesting area for future study.

The association of misframed proteins with AD and other disease states and the ability of hUbb^+1^ to inhibit proteasome activity in cultured cells in a dose-dependent manner is consistent with the idea that accumulation of misframed proteins may be detrimental to the aging animal. It will be important to determine if the increased abundance of misframed proteins in old flies is due to increased rates of MM, decreased clearance of the abnormal RNA species, decreased turnover of the misframed proteins, or some combination of these processes. Consistent with a toxic effect of accumulated protein damage during aging, old flies are more sensitive to proteasome inhibitors [60], and over-expression of certain enzymes implicated in protein repair such as protein carboxyl methyltransferase [61] and methionine sulfoxide reductase A [62] are reported to increase fly life span under appropriate conditions.

The fact that misframed proteins can have toxic effects and appear to increase in abundance during aging in mammals and in flies is consistent with an error catastrophe model, however other explanations exist. For example the apparently abundant expression of Ub^+1^ in young, wild-type flies may indicate a normal physiological function. Epigenetic regulation of gene expression and phenotypes is increasingly apparent across species [63]. Bistable switches are common and appear to allow phenotypic plasticity on various timescales [64]. Interestingly, repeated DNA sequence motifs are commonly associated with such epigenetic mechanisms. Stress response genes, particularly oxidative stress response genes such as heat shock proteins (hsps), are induced during normal aging of flies as well as in human aging-related disease states such as AD [11-14]. The genes encoding ubiquitin are induced in response to heat and oxidative stress in flies [37] and mammals [38], and perhaps MM represents an evolutionarily conserved epigenetic mechanism by which ubiquitin genes encode alternate proteins with differing functions expressed in response to certain physiological conditions. For example altered chromatin structure, altered RNA polymerase structure, or low nucleotide concentrations might each be predicted to increase rates of MM. The increased abundance of MM in old flies could conceivably represent a compensatory response with a benefit for continued function of cells or the animal. Consistent with this idea, in cultured mammalian cells the expression of hUbb^+1^ caused induction of hsp70 and increased resistance to oxidative stress [24]. Alternatively, even if MM might serve some conserved beneficial role earlier in the life cycle, such as in response to oxidative stress, its chronic activation during aging might be counterproductive. The ability to observe MM in the fly should allow us to begin to distinguish between these possibilities, and perhaps provide a model for studying the role of MM in human aging-related diseases.

## METHODS

### Plasmid construction

Transgenic constructs were generated by PCR amplification of insert fragments from plasmid templates, using primers engineered to create a PstI site at the 5' end and an EcoRI site and a polyadenylation signal sequence at the 3' end, and these fragments were cloned into the unique PstI and EcoRI sites of USC1.0 vector, as previously described [65]. All construct sequences were confirmed by sequencing. The hUbb and hUbb^+1^ constructs were generated using plasmid templates encoding the respective human sequences [20], and further details and oligo sequences are presented in Supplemental materials. The eGFP and DsRED Tet-on reporter constructs were generated in the USC1.0 vector, using the eGFP and DsRED gene sequences from the plasmids pGreen Pelican and pRHP, respectively [66]. The constructs were named TetO-GFP and TetO-DsRED respectively, and further details on their construction are provided in Supplemental materials.

### P element mediated transformation

Four independent germ-line transformants of the hUbb construct (hUbb [8], [118], [8] and [70]) were generated using standard methods [67]. All four lines integrated onto the 2^nd^ Chromosome. In addition, the hUbb[118] insert was mobilized using delta2-3 transposase source [56] to generate a strain with two copies of the insert, named hUbb[118D]. Six independent germ-line transformants were generated for the hUbb^+1^ construct. hUbb^+1^ [4], [1], and [11] integrated onto the 2^nd^ chromosome, while hUbb^+1^ [6], [30], and [19] integrated onto the 3^rd^ chromosome. Southern analysis indicated the presence of single inserts for each of the lines. Two independent germ-line transformants were generated for the TetO-GFP construct, lines TetO-GFP[21] and TetO-GFP[8], both inserted on third chromosome. Four lines were generated for the TetO-DsRED construct, lines [6] and [26B] on the third chromosome, and lines [1] and [21] on the second chromosome.

### Drosophila culture and life span assays

Drosophila were cultured on a standard agar/dextrose/corn meal/yeast media [68]. Unless otherwise indicated, “Young” flies were 10 days of age, and “Old” flies were 65 days of age. Where indicated, flies were cultured on food supplemented to a final concentration of 640μg/ml DOX for the experimental group [56]. Each of the indicated hUbb and hUbb^+1^ transgenic strains, as well as Oregon R wild-type flies (provided by Bloomington Drosophila stock center) and the w[1118] strain control were crossed to the “TO-daughterless” driver line, which contains the *daughterless*-GAL4 driver and the “901” bridge construct where a UAS-promoter drives expression of rtTA-M2alt [56, 69]. Crosses were performed at 25°C in urine specimen bottles. Prior to eclosion of the majority of pupae, bottles were cleared of adult parents and newly eclosed flies were allowed to emerge over the next 48 hours. Males and females each containing both the target transgene and the driver constructs were scored and collected. At day 4, the males and females were split into experimental and control groups. These were maintained at 29°C at 25 flies per vial. All flies were transferred every two days into fresh media for the first month and then every day for the following months. Additional life span assays were conducted at 25^0^C, and in these cases flies were transferred to fresh food every other day for the duration of the experiment. The number of dead flies was counted at each transfer and used to calculate mean and median life spans for the experimental (+DOX) and control (-DOX) groups. The statistical significance of the difference in median life span was calculated for each experiment using log rank tests in R statistical environment.

### Northern analyses

Each of the indicated hUbb and hUbb^+1^ transgenic strains and the Oregon R control strain were crossed to the rtTA(3)E2 driver line [55] and cultured at 25°C in urine specimen bottles. Males containing both the transgene and the rtTA(3)E2 driver were scored and collected. The males were then split into experimental and control group, each containing 100 flies. These were maintained at 25°C at 25 flies per vial. Flies were cultured on plus and minus DOX food for two weeks, and total RNA was isolated from 30 adult Drosophila males using the RNAqueous kit (Ambion), fractionated on 1.0% agarose gels and transferred to GeneScreen membranes (DuPont/NEN). 1X = 5 μg, and 2X = 10 μg. The PCR product UBBwt-1 was used as a specific probe for the hUbb gene. Blots were also hybridized with probe specific for ribosomal protein gene *Rp49* as a loading control [70]. DNA probes were 32P-labelled using the Prime-It II DNA labeling kit (Stratagene). Hybridization was carried out in Church-Gilbert solution at 65°C overnight. Hybridization signals were visualized and quantified using the phosphoimager and ImageQuant software (Molecular Dynamics).

### Developmental effects of hUbb and hUbb^+1^ overexpression

To quantify developmental survival, 4 virgins of the rtTA(3)E2 driver line were crossed to 4 males of the indicated transgenic strains, per vial. 4 replicate vials were set up with plus DOX food and 4 replicate vials with minus DOX food. Flies were cultured on food supplemented to a final concentration of 640μg/ml Doxycycline for the experimental group. The rtTA(3)E2 driver chromosome is balanced over the TM3 balancer chromosome, which is marked with the dominant mutation Sb. Therefore adult progeny marked with Sb contain the balancer chromosome and not rtTA, whereas the non-Sb progeny contain both rtTA and the target transgene, allowing for transgene over-expression in the presence of DOX. Reduced survival of flies over-expressing the trasngene is therefore indicated by the absence of non-Sb progeny. The resultant adult progeny were scored for the presence of the Sb marker, and the mean percent non-Sb flies is plotted, with error bars indicating the standard deviation across the 4 replicate vials. P values were generated using chi-square test in Excel.

### Western analyses

Several antibody reagents were purchased from Upstate cell signaling solutions, including Anti-Ubb (Catalog #07-375) and antibody specific for hUbb^+1^ (“Ubi2a”), both characterized previously [17]. For each of the lines, 30 flies from the experimental group (+DOX) and 30 flies from the control group (-DOX) were collected at 26 days (Time point 1), 48 days (Time point 2), 67 days (Time point 3), and 82 days (Time point 4) using brief CO_2_ anesthetization. The thirty adult flies were directly homogenized in Laemmli SDS sample buffer (Bio-Rad) in an attempt to maximize efficiency of protein extraction per fly and to minimize any possible protein degradation. The samples were boiled for 10 minutes, vortexed, cooled and fractionated on SDS-PAGE. Dilutions were made from the boiled supernatants. Unless otherwise indicated, the stacking gel was 4% and the running gel was 12%. The samples were transferred to nitrocellulose membrane (Bio-Rad) and the membrane was blocked overnight at 4°C in PBST supplemented with 5% Non-Fat Dry Milk (Bio-Rad). The nitrocellulose blots were incubated with 1:2000 of primary antibody specific to Ubb^+1^. The antibody diluent was made fresh each time in 1% BSA/PBST and incubated overnight at 4°C. Horseradish peroxidase-conjugated goat anti-rabbit secondary antibody (Amersham) was diluted to 1:3000 in 1% BSA/PBST and incubated at room temperature for 2 hours. After washing steps, the samples were briefly incubated in chemiluminescence reagent Plus (Perkin Elmer) and the bands were detected using Kodak Image Station. Quantitative differences in protein abundance between young and old samples and between plus and minus DOX samples were determined using Image J software, and were confirmed using multiple Westerns and by comparison to standard samples run in parallel.

## SUPPLEMETAL MATERIAL



## Abbreviations

hUbb: human ubiquitin-B
hApp: human amyloid precursor protein
hsp: heat shock protein
MM: molecular misreading
Ub: ubiquitin
