# Supplementary material for: Ubiquitin over-expression phenotypes and ubiquitin gene molecular misreading during aging in Drosophila melanogaster
Source: Aging (Albany NY). 2011 Mar 12;3(3):237–61. doi: 10.18632/aging.100278 (PMC3091519; doi:10.18632/aging.100278)
Supplement: Supplementary file 1 [file aging-03-237-s001.pdf]

SUPPLEMENTAL MATERIAL

SUPPLEMANTAL METHODS

hUbb and hUbb^{+1} constructs. To create the hUbb construct, intermediate PCR products (named UBBwt-1 and UBBwt-2) were obtained using a pcDNA3 vector containing the human UBBwt cDNA as a template. UBBwt-1 was generated using primers Uwt-1F (5' GGCTGCAGGAATTCGATATCAAGCT 3') and Uwt-1R (5' TTTATTAAGGCACAGTCGAGGCTGATCAG GCGA 3'). UBBwt-2 was generated using primers Uwt-2F (5' TGCAGGCTGCAGGAATTCGATATCAA GCT 3') and Uwt-2R (5' AATTTTTATTAAGGCACA GTCGAGGCTGATCAGCGA 3'). Both products were generated using pfu DNA polymerase (Stratagene). Products UBBwt-1 and UBBwt-2 were mixed together and boiled for 10 min at 95°C and cooled to room temperature to generate a reannealed UBBwt gene with a PstI site engineered at the 5' end and an EcoRI site at the 3' end. This fragment was cloned into the unique PstI and EcoRI sites of USC1.0 to generate the construct hUbb. The construct hUbb+1 was generated using the same type of procedure, and the intermediates were generated as follows: UBB+1-1 was generated using primers U+1-1F (5' GATCCATGCAGATCTTC GTGAAAAC 3') and U+1-1R (5' TTTATTCCAGTGT GATGGATATCTGCAGAAT 3'). UBB+1-2 was generated using primers U+1-2F (5' TGCAGATCCAT GCAGATCTTCGTGAAAAC 3') and U+1-2R (5' AATTTTTATTCCAGTGTGATGGATATCTGCAGA AT 3').

hApp and hApp^{+1} constructs and transgenic lines. hApp and hApp^{+1} constructs were generated using plasmid templates encoding the respective human sequences [1], using the same type of procedure as described above, and the intermediates were generated as follows: hAPPwt-1 was generated using primers Awt-1F (5' GTGCTGGAATTCTGCAGATATCCAT 3') and Awt-1R (5' TTTATTCGAGGTCGACGGTATCGATTCTT AA 3'). hAppwt-2 was generated using primers Awt-2F (5'TGCAGTGCTGGAATTCTGCAGATATCCAT 3') and Awt-2R (5' AATTTTTATTCGAGGTCGACGGT ATCGATTCTTAA 3'). hApp+1-1 was generated using primers A+1-1F (5' TAGAACTAGTGGATCCCCCG GGAGA 3') and A+1-1R (5' TTTATTCTCGTTGGCT GCTTCCTGTTCCAA 3'). hApp+1-2 was generated using primers A+1-2F (5'TGCATAGAACTAGTGGA TCCCCCGGGAGA 3') and A+1-2R (5' AATTTTTAT TCTCGTTGGCTGCTTCCTGTTCCAA 3'). Four independent germ-line transformants were generated for the hApp construct. hApp [16], [1] and [2] integrated onto the 2nd chromosome while hApp [24] integrated onto the 3rd chromosome. Four independent germ-line transformants were generated for the hApp^{+1} construct. hApp^{+1} [16] and [30] integrated onto the 2nd chromosome while hApp^{+1} [7] and [24] integrated onto the 3rd chromosome.

Tet-on eGFP and DsRED reporter constructs. For the eGFP reporter, PCR products were generated using pGreen Pelican plasmid containing the eGFP gene as a template. The coding region sequences were amplified using primers with a Pst1 site engineered at the 5' end and an EcoRI site engineered at the 3' end. The amplification products were then cloned into the unique PstI and EcoRI sites of USC1.0, to generate the final injection construct. The DsRED reporter construct was generated using the DsRED gene sequences from DsRED Pelican plasmid (pRHP) using analogous procedures.

hApp and hApp^{+1} Northern and Western analyses. The PCR product APPwt-1 was used as a specific probe for the hApp gene in Northern blot analyses. Western analysis of hApp and hApp^{+1} employed antibodies purchased from Upstate cell signaling solutions, including Anti-App (Catalog #07-667) as well as antibody specific for hApp^{+1} ("Amy-5") characterized previously [2]. Additional Western control experiments utilized mouse monoclonal antibody 22c11 (Millipore/Chemicon), specific for the N-terminus of hApp, and cortical neuron lysates as a positive control for App (data not shown).

SUPPLEMENTAL RESULTS

Analysis of hApp expression and molecular misreading

Human cDNA encoding wild-type hApp protein, and cDNA engineered with the appropriate dinucleotide deletions within the GAGAG motif were cloned downstream of the DOX-regulated promoter (Supplemental Figure S3A,B). These constructs were introduced into Drosophila using P element mediated transformation and multiple independent transgenic strains were generated for each construct. In all the experiments presented, the strains homozygous for the transgenic target constructs were crossed to the rtTA(3)E2 driver strain (or other driver strains, as indicated), to generate hybrid progeny containing both constructs; control flies contained only the rtTA(3)E2 driver construct and no target construct. Expression of hApp in adult male flies was assayed by Western blot, using a specific antibody (Upstate Cat. #07-667). No DOX-inducible species could be detected at the calculated size of ~79Kd, or at other sizes (Supplemental Figure S3D), suggesting that the hApp

protein is not being expressed at a detectable level and/or is not stable. Other studies have reported that hApp could be expressed in adult flies and detected by Western blot at an apparent MW of ~110Kd [3, 4]. One possibility is that hApp is being expressed at low levels in the experiments presented here, but is being obscured by a background band such as the one running at ~100Kd (Supplemental Figure S3D; indicated with asterisk). However DOX inducible expression of hApp was also not detected using mouse monoclonal antibody 22c11, which yielded a different pattern of background bands (data not shown). We conclude that hApp is either not being expressed at a detectable level from this construct in adult male flies, or that the protein is unstable. These hApp constructs are indeed being expressed in a DOX-dependent manner at the RNA level, as confirmed by Northern blots (Supplemental Figure S3C), and as indicated by the fact that they give rise to $hApp^{+1}$ via apparent MM events, as described next.

To determine if the misframed version of hApp could be detected in flies, Western blots were performed using antibody specific for $hApp^{+1}$. The $hApp^{+1}$ antibody readily detected His-tagged $hApp^{+1}$ protein purified from *E. coli* cells, as well as highly abundant protein produced in flies transgenic for the $hApp^{+1}$ transgenic construct at the same size, consistent with efficient expression of $hApp^{+1}$ in adult flies (Figure 5A; indicated by black arrowhead). Notably, both the His-tagged $hApp^{+1}$ and the $hApp^{+1}$ produced in transgenic flies ran in the gel at a position equivalent to an apparent MW of ~58Kd, which is the reported mobility for $hApp^{+1}$ under these conditions [5]. This is despite the fact that the calculated MW for the 348 amino acid residue $hApp^{+1}$ protein is ~39Kd. This unusual retarded mobility in SDS-PAGE gels observed for $hApp^{+1}$ (as well as hApp) has been observed in several previous studies [5, 6], and is attributed to the acidic region of the protein between positions 230-260 that contains many glutamate and aspartate residues. In transgenic flies expressing the hApp transgene, a DOX-inducible band at the same apparent MW of ~58KD was detected, consistent with MM of the hApp transgene (Supplemental Figure S4C, D). It is also interesting to note that there were several species in the Oregon-R control fly extracts that cross-reacted with $hApp^{+1}$ antibody, including one of a similar size as $hApp^{+1}$ (indicated by an asterisk), and that these species became more apparent with age (Supplemental Figure S4B). Despite this background, the fact that the apparently ~58Kd species was produced in a DOX-inducible manner in two independent hApp transgenic strains, but not in the controls, suggests that MM is indeed occurring, and moreover that this $hApp^{+1}$ protein is more readily detected in old flies.

The faint pattern of endogenous Drosophila species cross-reacting with the $hApp^{+1}$ antibody most likely represents non-specific, cross-reacting proteins, however it is not clear at this time why such cross-reactivity is more apparent in old fly extracts. The Drosophila genome contains at least one gene related to hApp, the *Appl* gene, however it is not obvious how it could encode a cross-reacting epitope or an appropriately sized protein based on its known sequence [3].

SUPPLEMENTAL REFERENCES

1. Gerez L, de Haan A, Hol EM, Fischer DF, van Leeuwen FW, van Steeg H, Benne R. Molecular misreading: the frequency of dinucleotide deletions in neuronal mRNAs for beta-amyloid precursor protein and ubiquitin B. Neurobiol Aging 2005; 26:145-155.

2. van Leeuwen FW, de Kleijn DP, van den Hurk HH, Neubauer A, Sonnemans MA, Sluijs JA, Koycu S, Ramdjielal RD, Salehi A, Martens GJ, Grosveld FG, Peter J, Burbach H, Hol EM. Frameshift mutants of beta amyloid precursor protein and ubiquitin-B in Alzheimer's and Down patients. Science 1998; 279:242-247.

3. Luo L, Tully T, White K. Human amyloid precursor protein ameliorates behavioral deficit of flies deleted for *Appl* gene. Neuron 1992; 9:595-605.

4. Greeve I, Kretzschmar D, Tschape JA, Beyn A, Brellinger C, Schweizer M, Nitsch RM, Reifegerste R. Age-dependent neurodegeneration and Alzheimer-amyloid plaque formation in transgenic Drosophila. J Neurosci 2004; 24:3899-3906.

5. Hol EM, van Dijk R, Gerez L, Sluijs JA, Hobo B, Tonk MT, de Haan A, Kamphorst W, Fischer DF, Benne R, van Leeuwen FW. Frameshifted beta-amyloid precursor protein (APP+1) is a secretory protein, and the level of APP+1 in cerebrospinal fluid is linked to Alzheimer pathology. J Biol Chem 2003; 278:39637-30643.

6. Weidemann A, Konig G, Bunke D, Fischer P, Salbaum JM, Masters CL, Beyreuther K. Identification, biogenesis, and localization of precursors of Alzheimer's disease A4 amyloid protein. Cell 1989; 57:115-126.

SUPPLEMENTAL FIGURES

A. hUbb construct sequence and transcript

+1 of transcript

tataaatagaggcgcttcgtctacggagcgacaattcaattcaaacaagcaaagtgaacacgtcgctaagcgaaagctaagc

aaataaacaagcgcagctgaacaagctaaacaatctgcaggctgcaggaattcgatatcaagcttatcgataccgtcgacctcg

Pst-I *
+86

agggggggccgccatgcagatcttcgtgaaaacccttaccggcaagaccatcacccttgaggtggagcccagtgacaccatc
gaaaatgtgaaggccaagatccaggataaggaaggcattcccccgaccagcagaggctcatctttgcaggcaagcagctgg
aagatggccgtactctttctgactacaacatccagaaggagtcgaccctgcacctggtcctgcgtctgagaggtggttaactcga
gggggggcccggtacccaattcgccctatagtgagtcgtattacgcgcgc**aataaa**aattc(EcoRI) *

Translation of frame 1

```
     N  S  I  Q  T  S  K  V  N  T  S  L  S  E  S  *  A  N  K  Q
1    aattcaattcaaacaagcaaagtgaacacgtcgctaagcgaaagctaagcaaataaacaa  60
     A  Q  L  N  K  L  N  N  L  Q  A  A  G  I  R  Y  Q  A  Y  R
61   gcgcagctgaacaagctaaacaatctgcaggctgcaggaattcgatatcaagcttatcga  120
     Y  R  R  P  R  G  G  A  A  M  Q  I  F  V  K  T  L  T  G  K
121  taccgtcgacctcgagggggggccgccatgcagatcttcgtgaaaacccttaccggcaag  180
     T  I  T  L  E  V  E  P  S  D  T  I  E  N  V  K  A  K  I  Q
181  accatcacccttgaggtggagcccagtgacaccatcgaaaatgtgaaggccaagatccag  240
     D  K  E  G  I  P  P  D  Q  Q  R  L  I  F  A  G  K  Q  L  E
241  gataaggaaggcattcccccgaccagcagaggctcatctttgcaggcaagcagctgg aa  300
     D  G  R  T  L  S  D  Y  N  I  Q  K  E  S  T  L  H  L  V  L
301  gatggccgtactctttctgactacaacatccagaaggagtcgaccctgcacctggtcctg  360
     R  L  R  G  G  *  L  E  G  G  P  G  T  Q  F  A  L  *  *  V
361  cgtctgagaggtggttaactcgagggggggcccggtaccc aattcgccctatagtgagtc  420
     V  L  R  A  Q  *  K
421  gtattacgcgcgcaataaaaatt  443
```

Translation of frame 2

```
     I  Q  F  K  Q  A  K  *  T  R  R  *  A  K  A  K  Q  I  N  K
2    attcaattcaaacaagcaaagtgaacacgtcgctaagcgaaagctaagcaaataaacaag  61
     R  S  *  T  S  *  T  I  C  R  L  Q  E  F  D  I  K  L  I  D
62   cgcagctgaacaagctaaacaatctgcaggctgcaggaattcgatatcaagcttatcgat  121
     T  V  D  L  E  G  G  P  P  C  R  S  S  *  K  P  L  P  A  R
122  accgtcgacctcgagggggggccgccatgcagatcttcgtgaaaacccttaccggcaa ga  181
     P  S  P  L  R  W  S  P  V  T  P  S  K  M  *  R  P  R  S  R
182  ccatcacccttgaggtggagcccagtgacaccatcgaaaatgtgaaggccaagatccagg  241
     I  R  K  A  F  P  P  T  S  R  G  S  S  L  Q  A  S  S  W  K
242  ataaggaaggcattcccccgaccagcagaggctcatctt tgcaggcaagcagctggaag  301
     M  A  V  L  F  L  T  T  T  S  R  R  S  R  P  C  T  W  S  C
302  atggccgtactctttctgactacaacatccagaaggagtcgaccctgcacctggtcctgc  361
     V  *  E  V  V  N  S  R  G  G  P  V  P  N  S  P  Y  S  E  S
362  gtctgagaggtggttaactcga gggggggcccggtacccaattcgccctatagtgagtcg  421
     Y  Y  A  R  N  K  N
422  tattacgcgcgcaataaaaatt  443
```

Supplemental Figure S1. Panel A

Translation of Frame 3 (Possible MM reading frame?)

```
     F  N  S  N  K  Q  S  E  H  V  A  K  R  K  L  S  K   *  T  S
3    ttcaattcaaacaagcaa agtgaacacgtcgctaagcgaaagctaagcaaataaacaagc  62
     A  A  E  Q  A  K  Q  S  A  G  C  R  N  S  I  S  S  L  S  I
63   gcagctgaacaagctaaacaatctgcaggctgcaggaattcgatatcaagcttatcgata  122
     P  S  T  S  R  G  G  R  H  A  D  L  R  E  N  P  Y  R  Q  D
123  ccgtcgacctcgagggggggccgccatgcagatcttcgtgaaaacccttaccggcaagac  182
     H  H  P  *  G  G  A  Q  *  H  H  R  K  C  E  G  Q  D  P  G
183  catcacccttgaggtggagcccagtgacaccatcgaaaatgtgaaggccaagatccagga  242
     *  G  R  H  S  P  R  P  A  E  A  H  L  C  R  Q  A  A  G  R
243  taaggaaggcattcccccccgaccagcagaggctcatctttgcaggcaagcagctggaaga  302
     W  P  Y  S  F  *  L  Q  H  P  E  G  V  D  P  A  P  G  P  A
303  tggccgtactctttctgactacaacatccagaaggagtcgaccctgcacctggtcctgcg  362
     S  E  R  W  L  T  R  G  G  A  R  Y  P  I  R  P  I  V  S  R
363  tctgagaggtggttaactcgagggggggcccggtacccaattcgccctatagtgagtcgt  422
     I  T  R  A  I  K  I
423  attacgcgcgcaataaaaatt  443
```

(The potential partial match to the Ubb[+1] epitope is in Red)

Supplemental Figure S1. Nucleotide sequences and translation of the transcripts expected from the transgenic constructs hUbb and hUbb[+1]. (**A**) The hUbb construct sequence and transcript. The sequence of the transgenic construct is presented starting from the TATA box of the promoter through the polyadenylation signal sequence (indicated in bold). The location of the unique PstI and EcoRI cloning sites of the USC1.0 vector are indicated by underline; the EcoRI site is destroyed during cloning. The location of nucleotide +1 of the transcript is indicated with an arrow. The coding region for wild-type ubiquitin is indicated in blue, and the stop codon is indicated in red with an asterisk. The translation of the entire transcript is presented in each of three reading frames. Methionine residues are indicated in blue, and stop codons are indicated with red asterisk. In translation frame 3, the potential partial match to the +1 epitope is indicated in red. (**B**) The hUbb[+1] construct sequence and transcript. The sequence of the transgenic construct is presented starting from the TATA box of the promoter through the polyadenylation signal sequence (indicated in bold). The location of the unique PstI and EcoRI cloning sites of the USC1.0 vector are indicated by underline; the EcoRI site is destroyed during cloning. The location of nucleotide +1 of the transcript is indicated with an arrow. The atg start codon for translation of the first Ubb repeat is indicated in blue bold-face, the corresponding atg sequence in the second repeat is indicated in blue. The gagag hotspot for MM is indicated with yellow highlight. The translation of the transcript is indicated below using single letter amino acid code. Note that this hUbb[+1] construct has been engineered to constitutively encode hUbb[+1] protein. This was done by deleting the conserved gt dinucleotide, located immediately downstream of the gagag hotspot, such that misframed translation proceeds into the second Ubb repeat to generate the +1 epitope, which is indicated in red.

B. hUbb[+1] construct sequence and transcript

tataaatagaggcgcttcgtctacggagcgacaattcaattcaaacaagcaaagtgaacacgtcgctaagcgaaagctaagc

Pst-I
+86

aaataaacaagcgcagctgaacaagctaaacaatctgcagatcc**atg**cagatcttcgtgaaaacccttaccggcaagaccatca
cccttgaggtggagcccagtgacaccatcgaaaatgtgaaggccaagatccaggataaggaaggcattcccccgaccagca
gaggctcatctttgcaggcaagcagctggaagatggccgtactctttctgactacaacatccagaaggagtcgaccctgcacctg
gtcctgcgtctgagagggtatgcagatcttcgtgaagaccctgaccggcaagaccatcaccctggaagtggagcccagtgaca
ccatcgaaaatgcgaaggccaagatccaggataaagaaggcatccctcccgaccagcagaggctcatctttgcaggcaagca
gctggaagatggctgcactttttctgactacaacatccagaaggagtcgaccctgcacctggtcctgcgtctgagaggtggtatg
cagatcttcgtgaagaccctgaccggcaagaccatcactctggaggtggagcccggtgacaccatcgaaaatgtgaaggccaa
gatccaagatagagaaggcatcccccccgaccagcagaggctcatctttgcaggcaagcagctggaagatggccgcactcttt
ctgactacaacatccagaaagagtcgaccctgcacctggtcctgcgcctgaggggtggctgttaagaattaattctgcagatatc
catcacactgg**aataaa**aattc(EcoRI)

```
     Y  K  *  R  R  F  V  Y  G  A  T  I  Q  F  K  Q  A  K   *  T
1    tataaatagaggcgcttcgtctacggagcgacaatt caattcaaacaagcaaagtgaaca  60
     R  R  *  A  K  A  K  Q  I  N  K  R  S  *  T  S  *  T  I  C
61   cgtcgctaagcgaaagctaagcaaataaacaagcgcagctgaacaagctaaacaatctgc  120
     R  S  M  Q  I  F  V  K  T  L  T  G  K  T  I  T  L  E  V  E
121  agatccatgcagatcttcg tgaaaacccttaccggcaagaccatcacccttgaggtggag  180
     P  S  D  T  I  E  N  V  K  A  K  I  Q  D  K  E  G  I  P  P
181  cccagtgacaccatcgaaaatgtgaaggccaagatccaggataaggaaggcattcccccc  240
     D  Q  Q  R  L  I  F  A  G  K  Q  L  E  D  G  R  T  L  S  D
241  gaccagcagaggctcatctttgcaggcaagcagctggaagatggccgtactctttctgac  300
     Y  N  I  Q  K  E  S  T  L  H  L  V  L  R  L  R  G   Y  A  D
301  tacaacatccagaaggagtcgaccctgcacctggtcctgcgtctgagagggtatgcagat  360
     L  R  E  D  P  D  R  Q  D  H  H  P  G  S  G  A  Q   *  H  H
361  cttcgtgaagaccctgaccggcaagaccatcaccctggaagtggagcccagtgacaccat  420
     R  K  C  E  G  Q  D  P  G  *  R  R  H  P  S  R  P  A  E  A
421  cgaaaatgcgaaggccaagatccaggataaagaaggcatccctcccgaccagcagaggct  480
     H  L  C  R  Q  A  A  G  R  W  L  H  F  F  *  L  Q  H  P  E
481  catctttgcaggcaagcagctggaagatggctgcactttttctgactacaacatccagaa  540
     G  V  D  P  A  P  G  P  A  S  E  R  W  Y  A  D  L  R  E  D
541  ggagtcgaccctgcacctggtcctgcgtctgagaggtggtatgcagatcttcgtgaagac  600
     P  D  R  Q  D  H  H  S  G  G  G  A  R  *  H  H  R  K  C  E
601  cctgaccggcaagaccatcactctggaggtggagcccggtgacaccatcgaaaatgtgaa  660
     G  Q  D  P  R  *  R  R  H  P  P  R  P  A  E  A  H  L  C  R
661  ggccaagatccaagatagagaaggcatcccccccgaccagcagaggctcatctttgcagg  720
     Q  A  A  G  R  W  P  H  S  F  *  L  Q  H  P  E  R  V  D  P
721  caagcagctggaagatggccgcactctttctgactacaacatccagaaagagtcgaccct  780
     A  P  G  P  A  P  E  G  W  L  L  R  I  N  S  A  D  I  H  H
781  gcacctggtcctgcgcctgaggggtggctgttaagaattaattctgca gatatccatcac  840
     T  G  I  K  I
841  actggaataaaaatt  855
```

Extrapolation to species <20Kd

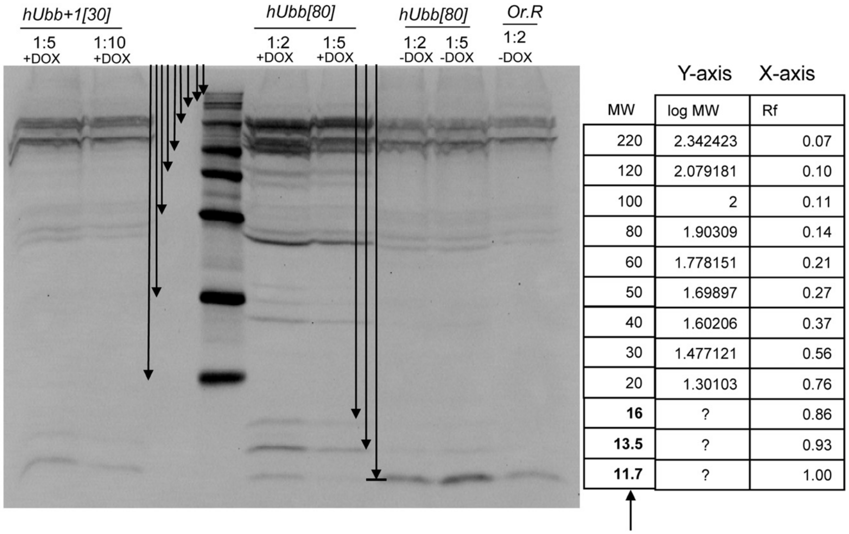

MW	Y-axis log MW	X-axis Rf
220	2.342423	0.07
120	2.079181	0.10
100	2	0.11
80	1.90309	0.14
60	1.778151	0.21
50	1.69897	0.27
40	1.60206	0.37
30	1.477121	0.56
20	1.30103	0.76
**16**	?	0.86
**13.5**	?	0.93
**11.7**	?	1.00

Y=-0.9061x + 1.9747

Y	10^y MW
1.195	**16**
1.132	**13.5**
1.068	**11.7**

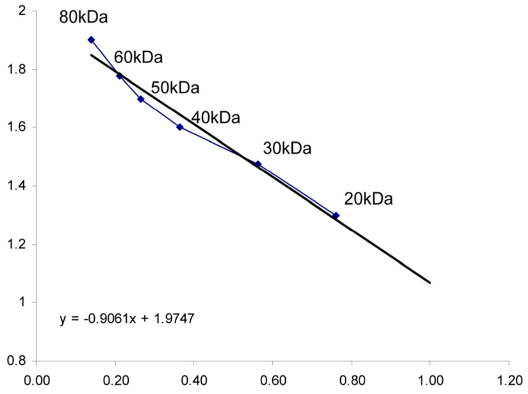

Explanation: Using equation from the linear regression line, the values for the Y-axis are calculated by plugging the values from X. Then the function 10^y generates back the MW of unknown protein.

Supplemental Figure S2. Estimation of apparent MW of various species recognized by hUbb^{+1} antibody.

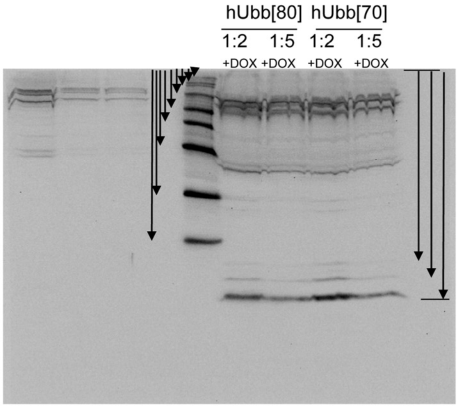

	Y-axis	X-axis
MW	log MW	Rf
220	2.342423	0.032895
120	2.079181	0.0625
100	2	0.072368
80	1.90309	0.108553
60	1.778151	0.180921
50	1.69897	0.240132
40	1.60206	0.355263
30	1.477121	0.5625
20	1.30103	0.756579
16.3	?	0.848684
14.23	?	0.917763
12.094	?	1

Y=-0.8601x + 1.9427	10^y MW
1.212747	16.3
1.153332	14.23
1.0826	12.094

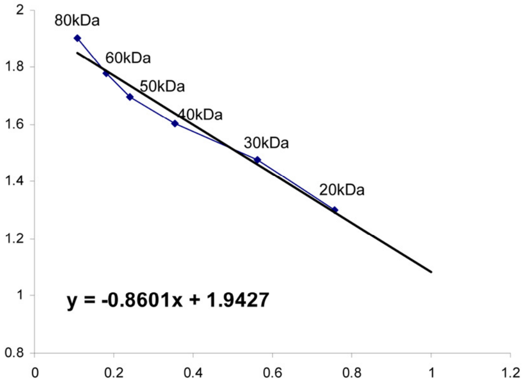

Explanation: Using equation from the linear regression line, the values for the Y-axis are calculated by plugging the values from X. Then the function 10^y generates back the MW of unknown protein.

Supplemental Figure S2

Ubb+1 conjugated to Ubbwt monomer(s)

Y=-0.8601(X) + 1.9427

MW	Log MW	Distance	Rf
**38.6**	1.587628	0.92	0.4461
**26.3**	1.419894	1.24	0.6078

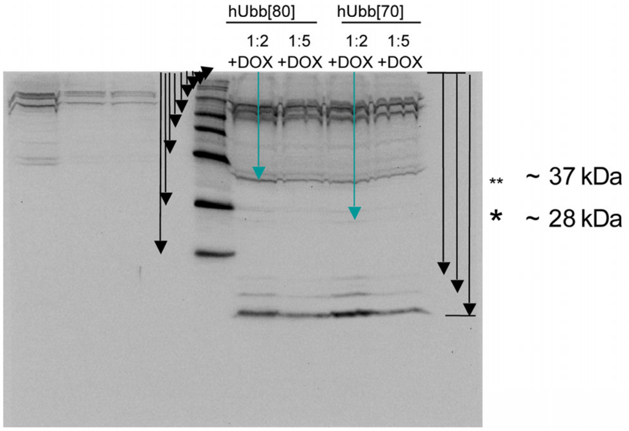

Ubb+1 conjugated to Ubbwt monomer(s) Blot 2

Y=-0.9061 (X) + 1.9747

MW	Log MW	Distance	Rf
37.78	1.577288	1.5	0.438596
26.9	1.43104	2.06	0.60

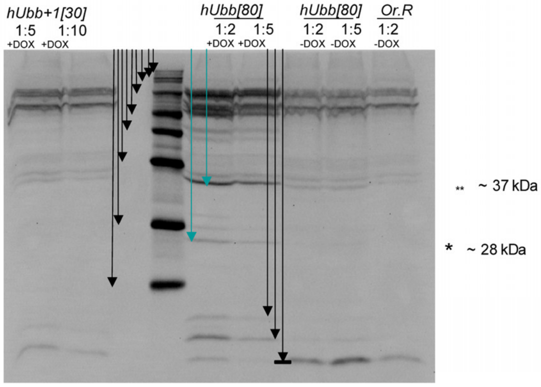

Supplemental Figure S2.

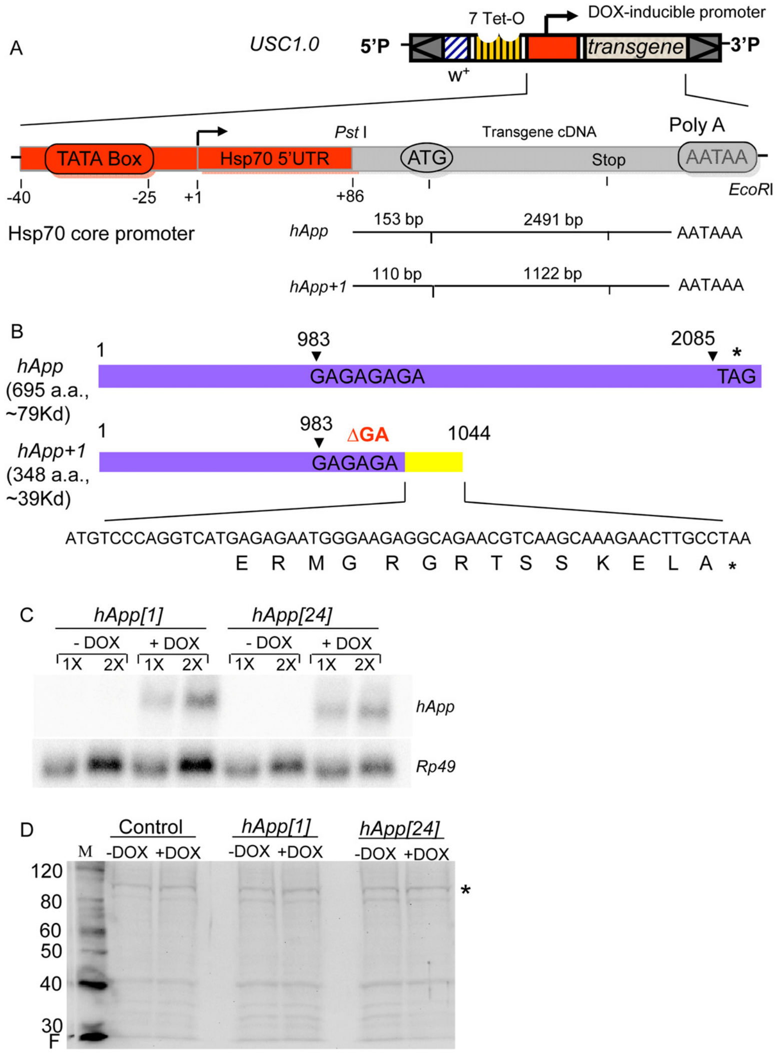

Supplemental Figure S3. hApp and hApp^{+1} transgenic constructs and conditional expression. (**A**) Diagram of hApp and hApp^{+1} constructs. The hApp and hApp^{+1} target constructs were generated by cloning the indicated cDNA fragments downstream of the DOX-inducible promoter in the USC1.0 vector between the unique *Pst*I and *EcoRI* sites. The number of bases present upstream and downstream of the A residue of the ATG start codon for normal translation are indicated for each cDNA insert. (**B**) Diagram of the sequence and reading frames of the hApp and hApp^{+1} constructs. The GAGAG hotspot is located in hApp exon 9. The amino acid sequence of the peptide used to generate the hApp^{+1} antibody is indicated using single-letter amino acid code. (**C**) Conditional hApp transgene expression. Flies of the indicated genotypes were cultured for one week on food supplemented +/- DOX, as indicated. Total RNA was fractionated and analyzed by Northern blot using probe specific for hApp, and probe for Rp49 as loading control. (**D**) Western analysis of hApp protein expression. Total protein was isolated from 30 male flies, fractionated using SDS-PAGE, Western blotted and incubated with antibody specific for hApp. The asterisk indicates an abundant endogenous cross-reacting protein migrating at a position corresponding to ~100KD.

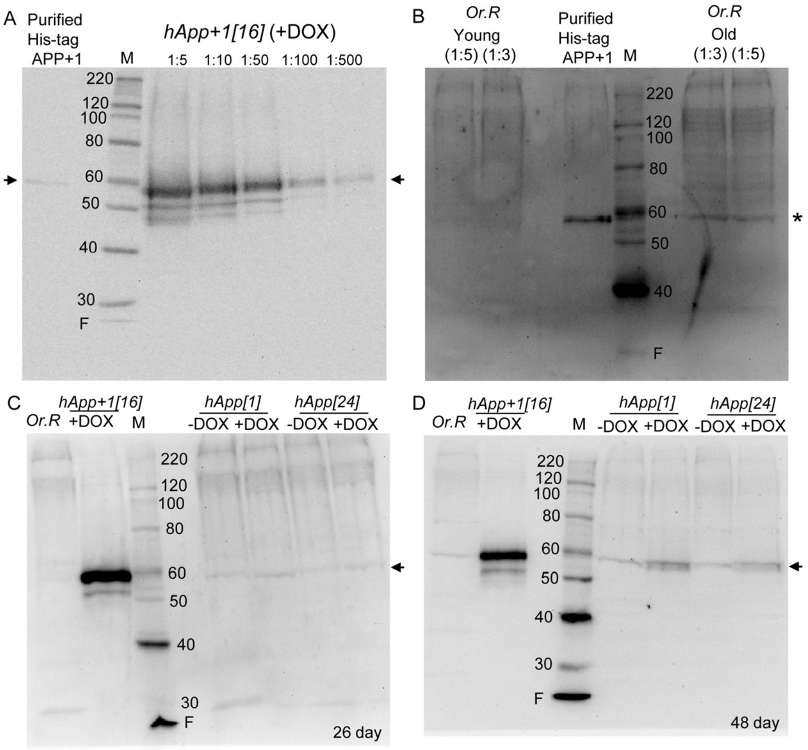

Supplemental Figure S4. Western blot analysis using antibody specific for hApp^{+1}. Total protein was isolated from 30 flies of the indicated genotypes, and assayed for the presence of protein that would be recognized by hApp^{+1} antibody; "young" is 10 days old and "old" is 65 days old. A. Molecular weight markers were run alongside His-tagged hApp^{+1} purified from *E. coli* cells, as well as the indicated dilutions of total protein isolated from adult flies in which the hApp^{+1} transgenic construct was expressed. B. Purified His-tagged hApp^{+1} protein from *E. coli* was run alongside protein from young and old Oregon-R (Or.R) control flies. C. Flies cultured +/- DOX for 26 days. D. Flies cultured +/- DOX for 48 days.
